# Constraining Evolution of *Alternaria alternata* Resistance to a Demethylation Inhibitor (DMI) Fungicide Difenoconazole

**DOI:** 10.3389/fmicb.2019.01609

**Published:** 2019-07-10

**Authors:** Meng-Han He, Yan-Ping Wang, E-Jiao Wu, Lin-Lin Shen, Li-Na Yang, Tian Wang, Li-Ping Shang, Wen Zhu, Jiasui Zhan

**Affiliations:** ^1^State Key Laboratory of Ecological Pest Control for Fujian and Taiwan Crops, Fujian Agriculture and Forestry University, Fuzhou, China; ^2^Key Laboratory for Biopesticide and Chemical Biology, Ministry of Education, Fujian Agriculture and Forestry University, Fuzhou, China; ^3^Fujian Key Laboratory of Plant Virology, Institute of Plant Virology, Fujian Agriculture and Forestry University, Fuzhou, China; ^4^Department of Forest Mycology and Plant Pathology, Swedish University of Agricultural Sciences, Uppsala, Sweden

**Keywords:** *Alternaria alternata*, population genetics, difenoconazole tolerance, fitness penalties, genetic variation

## Abstract

Evolution of fungicide resistance in plant pathogens is one of major concerns in sustainable plant disease management. In this study, the genetics and potential of developing resistance to a demethylation inhibitor (DMI) fungicide, difenoconazole, in the fungal pathogen *Alternaria alternata* was investigated using a comparative analysis of genetic variation in molecular (Single Sequence Repeats, SSR) and phenotypic (fungicide tolerance) markers. No difenoconazole resistance was found in the 215 *A. alternata* isolates sampled from seven different ecological zones in China despite the widespread use of the fungicide for more than 20 years. This result suggests that the risk of developing resistance to difenoconazole in *A. alternata* is low and we hypothesize that the low risk is likely caused by fitness penalties incurred by resistant mutants and the multiple mechanisms involving in developing resistance. Heritability and plasticity account for ∼24 and 3% of phenotypic variation, respectively, indicating that genetic adaptation by sequence variation plays a more important role in the evolution of difenoconazole resistance than physiological adaptation by altering gene expression. Constraining selection in the evolution of *A. alternata* resistance to difenoconazole was documented by different patterns of population differentiation and isolate-by-distance between SSR markers and difenoconazole tolerance. Though the risk of developing resistance is low, the findings of significant differences in difenoconazole tolerance among isolates and populations, and a skewing distribution toward higher tolerance suggests that a stepwise accumulation of tolerance to the fungicide might be occurring in the pathogen populations. As a consequence, dynamic management programs guided by evolutionary principles such as spatiotemporal rotations of fungicides with different modes of action are critical to prevent the continued accumulation of tolerance or the evolution of resistance to difenoconazole and other DMI fungicides.

## Introduction

Plant diseases caused by pathogenic fungi have been and continue to be one of the major factors threatening global food security and social stability and development (Brent and Hollomon, 2007; Savary et al., 2012; Chourasiya et al., 2013; Hahn, 2014). Outbreaks of several plant diseases in history such as the Irish Potato Famine, Bengal Rice Famine and the southern corn leaf blight pandemic in United States not only caused severe economic losses, but in the case of the first two diseases, also the death of millions of people from starvation (Strange and Scott, 2005). To counter such losses and human casualty, fungicides have been an essential weapon in the disease control armory especially when genetic controls fail. The application of fungicide not only benefits society directly and immediately by reducing food losses but also indirectly and in the long term, by improving the quality and longevity of human life and supporting economic development (Cooper and Dobson, 2007).

Due to their short generation time and large population size (Angelini et al., 2015; Delmas et al., 2017), fungal pathogens can rapidly evolve and adapt to meet changing environments including those imposed by the introduction of new fungicides in agricultural systems. Indeed, many fungicides have been rendered ineffective by the development of resistance in target pathogens (Avenot et al., 2016; Rupp et al., 2016; Delmas et al., 2017; Jørgensen et al., 2018). Resistant pathotypes usually originate from mutation and/or recombination (sexual and asexual) in a single population, increase frequency through natural selection and migrate to other populations directly or via intermediary stepping-stone populations (Gisi et al., 2002; Angelini et al., 2015).

Development of fungicide resistance is a complex and multifactorial process. It depends on many factors including: (i) properties of the fungicides such as their chemistry and mode of action, (ii) characteristics of the pathogens such as their reproductive rate and genetic adaptability, and (iii) ecological features such as host traits and environmental conditions (Brent and Hollomon, 2007; Damicone and Smith, 2009; Hahn, 2014; Muneret et al., 2018). In terms of their mode of action, fungicides can be either site-specific or site non-specific. Resistance to site-specific fungicides is a qualitative trait and can be developed by single point mutations occurring at the target sites. In this class of fungicides, resistant pathotypes can rapidly emerge in pathogen populations shortly after the fungicides are commercialized. For example, resistance to Quinone outside inhibitors (QoI) emerged in *Blumeria graminis* f. sp. *tritici* only 2 years after the fungicides were commercialized (Sierotzki et al., 2000; Villa et al., 2017). On the other hand, resistance to site non-specific fungicides is quantitatively inherited and involves a series of changes in genes governing the uptake, transport, storage and metabolism of the fungicide (de Waard et al., 2006). Therefore, the risk of developing resistance to this class of fungicides is expected to be lower than the risk involved with the use of site-specific fungicides.

Human activities also play an important role in the evolution of fungicide resistance, mainly through their influences on the direction and intensity of natural selection acting upon pathogens. Continuous and widespread application of fungicides with the same mode of action creates strong, directional selection for resistant mutants, thereby facilitating the development of fungicide resistance in pathogen populations. On the other hand, mixture or rotation of fungicides with different modes of action generates disruptive selection that reduces the probability of emergence of resistant mutants – a strategy that enhances the durability of fungicide efficacy (Brent and Hollomon, 2007).

Difenoconazole, approved in the 1988 in Europe (Bowyer and Denning, 2014), is synthesized as a novel demethylation inhibitor (DMI) fungicide, targeting sterol 14α-demethylase (CYP51), an important regulatory enzyme in the ergosterol biosynthetic pathway (Zarn et al., 2003; Price et al., 2015). The fungicide has been used worldwide in agricultural system due to its excellent, fast-acting and prominent systemic activity (Dong et al., 2013; Ge et al., 2017). It has both protective and curative efficacy and is typically used to control a broad spectrum of foliar, seed- and soil-borne diseases caused by Ascomycetes, Basidiomycetes. Deuteromycetes and oomycetes (Horsfield et al., 2010; Gveroska, 2013; Elansky et al., 2016). Resistance to difenoconazole is a polygenic trait with multiple mechanisms contributing to the final phenotype (Wyand and Brown, 2005; Leroux and Walker, 2011; Cools and Fraaije, 2013; Villani et al., 2015; Pereira et al., 2017). Possibly due to the intensive application of difenoconazole, isolates with reduced sensitivity have been detected in *Phoma ligulicola* (Jones et al., 2007) and *Venturia inaequalis* (Stevic′ et al., 2010). Cross-resistance was also found between difenoconazole and other DMI fungicides such as myclobutanil, fenbuconazole, and flusilazole in the pathogen *V. inaequalis* (Stevic′ et al., 2010; Pfeufer and Ngugi, 2012).

Early blight is a destructive foliar disease reducing the photosynthetic ability of potato, tomato and other important Solanaceae crops in the world (Gent and Schwartz, 2003; MacDonald et al., 2007; Meng et al., 2015b). It can cause significant economic loss to farmers when environmental conditions (e.g., nitrogen and water shortages’) favor for epidemics. The causal agents of early blight are haploid filamentous fungi from the genus *Alternaria* (Parvez, 2003; Leiminger and Hausladen, 2012). In China, *Alternaria alternata* has replaced *A. solani* as the main pathogen inducing potato early blight (Meng et al., 2015b; Zheng et al., 2015). It is a global pathogen dispersed by rain-splash, wind or infected plant materials (Reis et al., 2006). Although no sexual fruiting bodies have been documented yet, population genetic and phylogenetic analyses suggest that cryptic sex may occur frequently in the life-cycle of the pathogen (Meng et al., 2015a). Besides potato early blight, *A. alternata* can also cause catastrophic diseases on other numerous food and ornamental crops (Wier et al., 1998; Woudenberg et al., 2013) although host specificity has been reported in the pathogen (Elena, 2006; Woudenberg et al., 2015). In potato industry, cultivars with major-gene resistance to *A. alternata* are rare and management of early blight is mainly relied on the application of fungicides. Difenoconazole has been used to battle against *A. alternata* for many years around the world including China (Zheng et al., 2013; Avenot et al., 2016; Fonseka and Gudmestad, 2016; Wang et al., 2016).

Knowledge of genetic and evolutionary mechanisms responsible for the development of fungicide resistance in pathogen populations is important as a basis for formulating effective approaches to plant disease management. This knowledge can be acquired through statistical analyses of genetic variation and its spatial dynamics in fungicide sensitivity (Qin et al., 2016). Therefore, the main objectives of the present study were to: (i) evaluate the potential for developing difenoconazole resistance in *A. alternata*, by quantifying genetic and environmental factors contributing to phenotypic variation of difenoconazole tolerance using a common garden experiment and (ii) determine the role of natural selection in the evolution of difenoconazole resistance by comparing the spatial distribution of genetic variation in SSR markers and difenoconazole tolerance.

## Materials and Methods

### Origin of *Alternaria alternata* Isolates

Seven *A. alternata* populations with a total of 215 genetically distinct isolates from potato host were included in the current analysis of difenoconazole tolerance. The isolates were sampled during the 2011 and 2012 potato growing seasons from seven fields distributed across various ecological niches and agro-ecosystems of China including Fujian (FJN), Heilongjiang (HLJ), Henan (HNN), Hubei (HBI), Inner Mongolia (IMG), Shandong (SDG), and Yunnan (YNN). They were collected from plants separated by >100 cm and The *A. alternata* isolates were molecularly assayed with eight microsatellite markers (*PAS1*, *PAS2*, *PAS3*, *PAS4*, *PAS5*, *PAS6*, *PAS7*, and *Ad8*) previously and genotypes of the isolates were determined by GenClone 2.0 (Arnaud-Haond et al., 2007) using the allele information at each of the eight SSR loci. Detailed information on pathogen collection, isolation, DNA extraction and microsatellite genotyping as well as primer sequences can be found in these previous publications (Benichou et al., 2009; Meng et al., 2015a,b). Because potato early blight can be induced by several species in the Alternaria genus, the identity of these isolates were checked morphologically by spore characterization under a light microscope (Meng et al., 2015a, 2015b) and molecularly by PCR amplifications of ITS (Internal transcribed spacer) regions and histone 3 gene (Meng et al., 2015a; Zheng et al., 2015) to ensure they are all *A. alternata*.

### Measurement of Difenoconazole Tolerance

A total of 215 isolates were tested for difenoconazole tolerance by a growth rate assay using a common garden design (Moloney et al., 2009; Yang et al., 2016; He et al., 2018). The pathogen isolates taken out from long-term storage were activated on PDA plates for 6 days. Mycelial plugs (ϕ = 5 mm) taken from the edge of the colony were transferred to fresh PDA plates with or without amendment of 0.02, 0.06, or 0.12 μg/mL of difenoconazole (technical grade). Preliminary experiments indicated these concentrations provided the best resolution with the least experimental error. Many isolates did not grow at a higher difenoconazole concentration while growth rates of these isolates were unchanged at a lower concentration. The PDA plates inoculated with different *A. alternata* isolates were divided into three groups each corresponding to one difenoconazole concentration and arranged according to a completely randomized design with three replicates in a single incubator set to 24°C. Pathogen inoculated on the PDA plates without difenoconazole ingredient was set as a control for each isolate and controls were included in each fungicide concentration. To minimize experimental errors, plate preparation, pathogen inoculation and colony measurement for the entire fungicide tolerance assay were completed by the same student with all experimental activities for a single fungicide concentration being assessed on the same day. Pathogen colonies were digitalized daily between 2nd and 6th post-inoculation and Assess (Lamari, 2002) was used to estimate the size (area) of the colonies. In total, 12900 [215 isolates × 3 replicates × 4 treatment (3 fungicide concentrations + 1 control) × 5 measurements] colonies were measured, generating a large number of data points to evaluate the spatial pattern, adaptive mechanism and evolutionary history of difenoconazole resistance in the *A. alternata* populations.

### Data Analysis

Intrinsic growth rate of the pathogen isolates was estimated as described previously (Qin et al., 2016; Yang et al., 2016; He et al., 2018). A logistic model was applied for the estimate (Aguayo et al., 2014) using the daily colony sizes of isolates measured over the 6 days of inoculation and the intrinsic growth rate was estimated separately for each of the three difenoconazole concentrations. The colony size of the pathogen isolates at the first day of inoculation was set to 0.2 cm^2^ (πr^2^ = 3.14 × 0.25^2^). the size of the mycelial plug initiated the colonies and the capacity of colony growth (*K*, the maximum colony size) for the logistic model was set to 63.6 cm^2^ (πr^2^ = 3.14 × 4.5^2^), the area of a Petri dish with a diameter of 9 cm. Difenoconazole tolerance was measured by the relative intrinsic growth rate (RGR) of the pathogen isolates in the presence to the absence of difenoconazole (Zhan et al., 2006; Brunner et al., 2016; He et al., 2018). The percentile of difenoconazole tolerance in the combined population of the 215 *A. alternata* isolates was tabulated using a bin system as described previously (Qin et al., 2016; Wu et al., 2019). General linear model procedure was used to evaluate the contribution of population, fungicide concentration, pathogen genotype and genotype-concentration interaction to the phenotypic variance of difenoconazole tolerance and least significant difference (Kokalisburelle et al., 2013) was used to determine the spatial variation of difenoconazole tolerance in the *A. alternata* populations from different locations.

Single Sequence Repeats data of the 215 isolates were taken from a previous study (Meng et al., 2015a) and used to estimate Nei’s gene diversity, population differentiation *G*_ST_ and the effective number of migrants (*N_e_m*) in neutral markers (Nei, 1973) using Popgene 3.249 (Yeh et al., 2000). Phenotypic variance in difenoconazole tolerance was partitioned into sources attributable to isolate (*I*, random effect), population (*P*, random effect), and fungicide concentration (*C*, fixed effect) using SAS GLM and VARCOMP programs (SAS 9.4, SAS Institute) according to the model:

(1) $$Y_{\mathrm{ripc}}=M+I\,\left(P\right)+C+P+I\,\left(P\right)\times C+P\times C+E_{\mathrm{ripc}}$$

Where *Y*_ripc_ refers to the mean RGR of replicate *r* for isolate *i* in population *p* at concentration *c*; *M*, *P*, *I*(*P*), *I*(*P*) × *C*, *P* × *C* and *E*_ripc_ refer to the overall population mean, genetic variance among populations, genetic variance within populations, variance due to genotype × concentration interaction, variance responses of populations to dose effect and the variance among replicates, respectively (Zhan and McDonald, 2011). In common garden experiments with asexual species, any among-replicate variation in the phenotypic value of an isolate can be treated as environmental effect. Therefore, variance among replicates in this case is equivalent to the environmental variance of RGR (Zhan et al., 2005). Population differentiation (*Q*_ST_) in difenoconazole tolerance as measured by RGR was estimated in a similar way to the estimation of population differentiation of SSR marker loci (*G*_ST_) using the formula described in previous publications (Qin et al., 2016; Yang et al., 2016).

Heritability of RGR in a population was estimated by dividing genetic variance within populations by total phenotypic variance and phenotypic plasticity was calculated by dividing the variance of isolate-concentration interaction by the total phenotypic variance (Tonsor et al., 2013). Statistical difference between the overall *G*_ST_ in SSR loci and overall *Q*_ST_ in fungicide sensitivity was tested using the standard deviation of *Q*_ST_ constructed from 100 resamplings of the original data (Zhan and McDonald, 2011). Difenoconazole tolerance measured by RGR among *A. alternata* populations from collection sites were compared by Least significant difference (LSD) test (Ott and Longnecker, 2008). Physical distance between collection sites was estimated by Google Earth. Isolation-by-distance in *A. alternata* was inferred by association analysis between Napierian logarithm of the pair-wise physical distance and pair-wise gene flow among the pathogen populations, and association of fungicide resistance (RGR) among difenoconazole doses was evaluated by Pearson’s correlation (Lawrence and Lin, 1989).

## Results

### Frequency Distribution of *Alternaria alternata* Tolerance (RGR) to Difenoconazole

**FIGURE 1:**
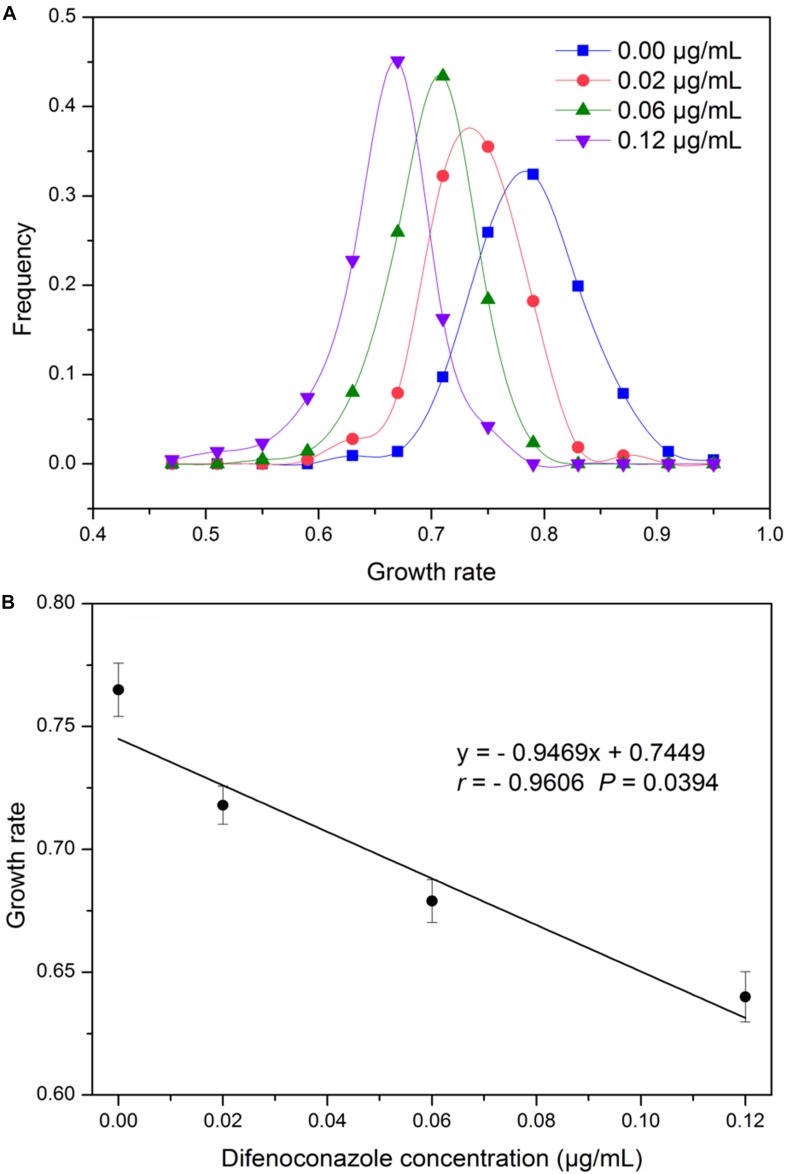
Growth rate of *Alternaria alternata* collected from seven potato fields across China: **(A)** distribution of growth rate at in the presence and absence of difenoconazole, **(B)** correlation between difenoconazole concentrations and average growth rate of *A. alternata* populations collected from seven potato fields across China.

Between 28 and 32 (total 215) genetically distinct *A. alternata* isolates originating from seven field populations were assayed for growth rate in the absence and presence of difenoconazole. The growth rate of the 215 *A. alternata* isolates displayed a continuous, unimodal distribution both in the absence and presence of difenoconazole (Figure 1A). The average growth rate of colonies declined as the concentration of the fungicide increased (*r* = −0.96, *P* = 0.04; Figure 1B). In the absence of the fungicide, the average growth rate was 0.765 cm^2^/day. This value declined linearly to 0.640 cm^2^/day at a fungicide concentration of 0.12 μg/mL.

### Distribution of Difenoconazole Tolerance Measured by RGR Between Presence and Absence of the Fungicide

**FIGURE 2:**
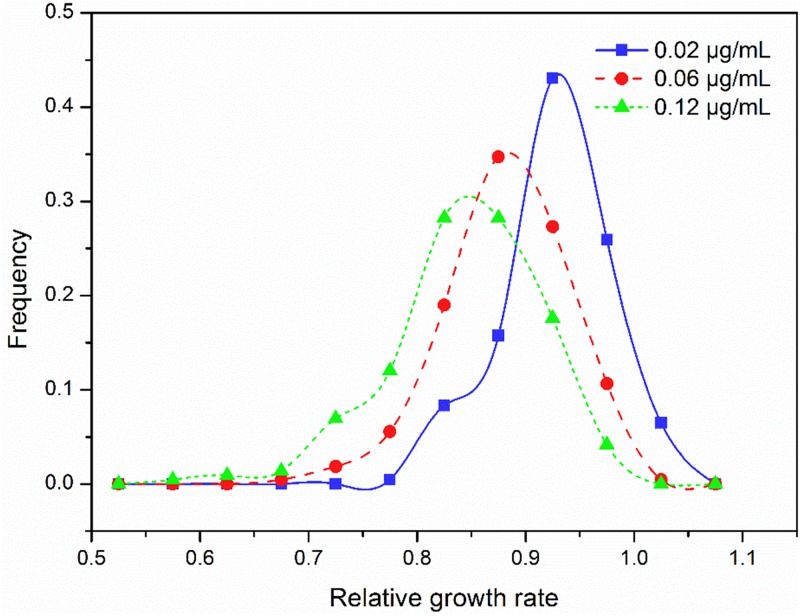
Frequency distribution of difenoconazole tolerance measured by the relative growth rate in the presence to the absence of difenoconazole at three concentrations in the 215 isolates of *Alternaria alternata* collected from seven potato fields across China.

**FIGURE 3:**
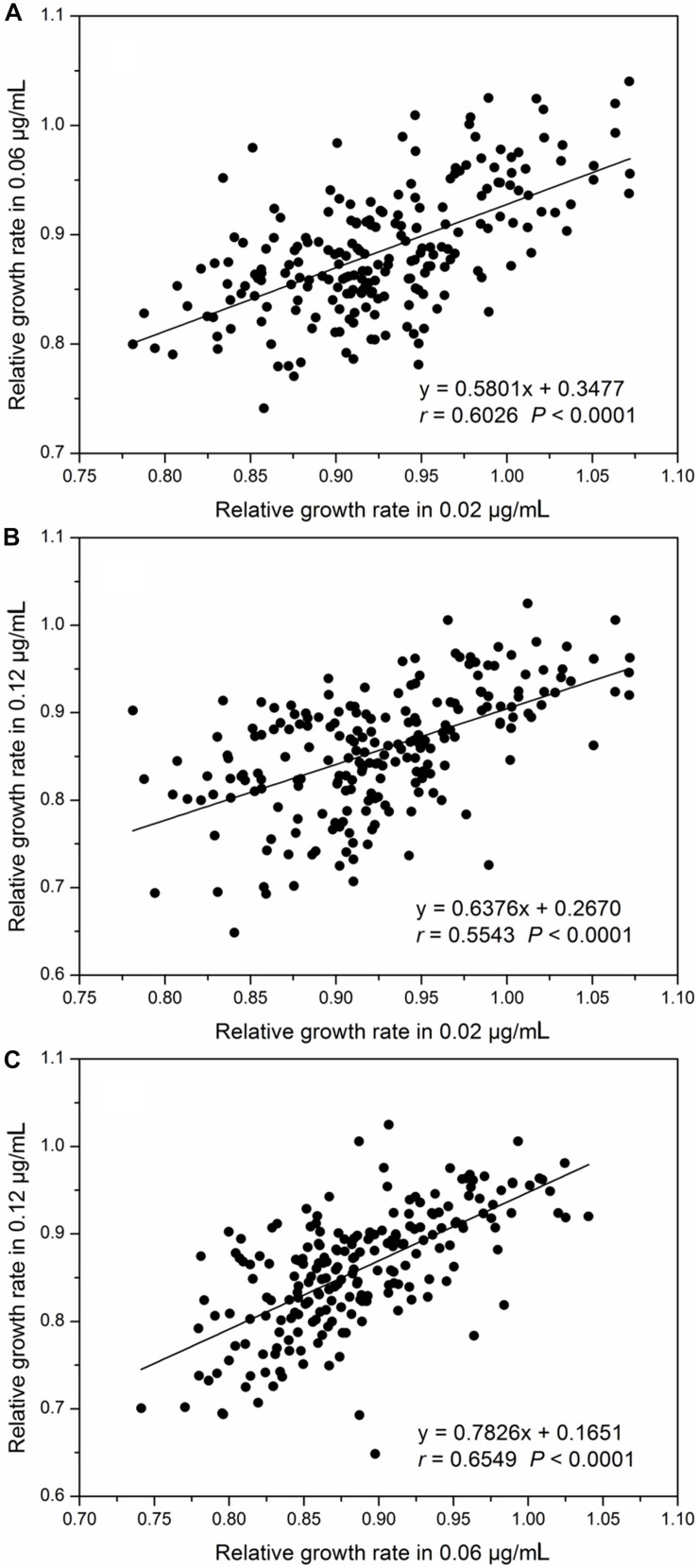
Correlation between relative growth rates (difenoconazole tolerance) in different concentrations: **(A)** difenoconazole concentrations at 0.02 and 0.06 μg/mL, **(B)** difenoconazole concentrations at 0.02 and 0.12 μg/mL, and **(C)** difenoconazole concentrations at 0.06 and 0.12 μg/mL.

Relative intrinsic growth rate of the 215 isolates also displayed a continuous, unimodal distribution in all three concentrations (Figure 2), ranging from 0.74 to 1.07 with an average of 0.92 at the 0.02 μg/mL, 0.63 to 1.04 with an average of 0.88 at the 0.06 μg/mL and 0.53 to 1.02 with an average of 0.85 at the 0.12 μg/mL, respectively. As fungicide concentration increased, the mean RGR of *A. alternata* populations decreased but the ratio of RGR in the fastest and slowest growth isolates increased. In the 0.02 μg/ml treatment, the ratio of RGR between the fastest and slowest growth isolates in the population was 1.44. This value increased to 1.66 and 1.92 for the 0.06 and 0.12 μg/mL treatments, respectively. Association analysis showed that *A. alternata* tolerance in different concentrations was positively and significantly correlated (*P* < 0.0001, Figure 3).

### Spatial Variation in Difenoconazole Tolerance (RGR) Among *Alternaria alternata* Populations

Population, isolate and fungicide dose all contributed significantly (*P* < 0.0001) to the performance of difenoconazole tolerance in *A. alternata* as measured by RGR (Table 1). The 215 *A. alternata* isolates also responded differentially to different concentrations. Least significant difference (LSD) analysis indicated that the pathogen population sampled from Shandong showed the least tolerance to difenoconazole while the pathogen population sampled from Heilongjiang was most tolerant to difenoconazole (Table 2). The populations sampled from Hubei and Inner Mongolia showed an intermediate level of tolerance (Table 2).

**TABLE 1:** Analysis of variance (ANOVA) of difenoconazole tolerance in the 215 isolates of *Alternaria alternata* sampled from seven potato fields in China.

**Parameter**	**Source**	**DF**	**SS**	**Mean SS**	***F*-value**	***P***
RGR	Population	6	1.53	0.255	27.73	<0.0001
	Concentration	2	3.89	1.947	211.78	<0.0001
	Isolate	209	19.79	0.095	10.3	<0.0001
	Concentration ^*^ Isolate	430	5.63	0.013	1.42	<0.0001
	Error	4759	42.03	0.009		

**TABLE 2:** Difenoconazole tolerance at three concentrations and mean of three concentrations in the 215 isolates of *Alternaria alternata* collected from seven potato fields across China.

**Population**	**Difenoconazole tolerance**			
	
	**0.02 μg/mL**	**0.06 μg/mL**	**0.12 μg/mL**	**Mean**
FJN	0.940⁢*AB*	0.895⁢*AB*	0.879⁢A	0.905⁢A
HBI	0.914⁢*CD*	0.880⁢*BC*	0.847⁢*BC*	0.881⁢*BC*
HLJ	0.946⁢A	0.904⁢A	0.886⁢A	0.912⁢A
HNN	0.939⁢*AB*	0.895⁢*AB*	0.880⁢A	0.905⁢A
IMG	0.924⁢*BC*	0.882⁢*BC*	0.85⁢*AB*	0.887⁢B
SDG	0.901⁢D	0.872⁢C	0.836⁢C	0.870⁢D
YNN	0.916⁢*CD*	0.873⁢C	0.834⁢C	0.874⁢*CD*

### Genetic Variations in RGR and SSR Marker Loci

The contribution of genetic architecture and gene expression to difenoconazole tolerance in the pathogen populations was measured by heritability and phenotypic plasticity. Heritability in the seven populations ranged from 0.17 to 0.47 with an average of 0.24; while phenotypic plasticity in the seven populations ranged from 0 to 0.17 with an average of 0.03 (Table 3). Heritability was higher than phenotypic plasticity in all seven populations. The *A. alternata* population collected from Yunnan displayed the highest heritability in difenoconazole tolerance while that collected from Hubei displayed the lowest heritability (Table 3). The average SSR diversity in the seven *A. alternata* field populations ranged from 0.31 to 0.62 with an overall diversity of 0.44 when the isolates from the seven populations were pooled (Table 3). The highest SSR variation was found in the *A. alternata* population sampled from Fujian while that the lowest SSR variation was found in the *A. alternata* population collected from Yunnan. No association (*r* = −0.49, *P* = 0.26) was detected between heritability of difenoconazole tolerance and genetic variation in SSR markers.

**TABLE 3:** Sample size, gene diversity in SSR marker loci, quantitative genetic parameters related to the level of tolerance to difenoconazole in the seven *Alternaria alternata* populations from potato.

**Population**	**Sample size**	**Gene diversity**	**Fungicide tolerance**		
			**Heritability**	**Plasticity**	R^*^
FJN	28	0.62	0.206	0.005	45
SDG	30	0.4	0.266	0.022	12
HBI	30	0.39	0.174	0.078	2
HNN	32	0.37	0.291	0.021	14
YNN	31	0.31	0.47	0.167	3
IMG	33	0.39	0.196	0.03	6
HLJ	31	0.36	0.23	0	∞
Overall	215	0.44	0.237	0.033	7

^*^Ratio of heritability to plasticity.

### Comparison of *Q*_ST_ in Difenoconazole Tolerance (RGR) and *G*_ST_ SSR Marker Loci

Pair-wise *Q*_ST_ in difenoconazole tolerance ranged from 0.00 to 0.12 and pair-wise *G*_ST_ in SSR markers ranged from 0.01 to 0.15. Thirteen out of twenty-one pairs of *G*_*ST*_ were higher than *Q*_ST_ (Table 4). The overall *G*_ST_ (0.12) in the SSR markers across the seven pathogen populations was significantly higher than overall *Q*_ST_ (0.028) in difenoconazole tolerance.

**TABLE 4:** Pair-population differentiation of SSR marker loci (*G*_ST_) and difenoconazole tolerance (*Q*_ST_) among the seven populations of *Alternaria alternata* sampled from potato.

	**FJN**	**SDG**	**HBI**	**HNN**	**YNN**	**IMG**	**HLJ**
FJN	–	0.1213	0.1095	0.1214	0.1143	0.1535	0.1440
SDG	0.0505	–	0.0055	0.0056	0.0084	0.0248	0.0081
HBI	0.0254	0.0000	–	0.0062	0.0180	0.0254	0.0129
HNN	0.0000	0.0488	0.0263	–	0.0169	0.0334	0.0116
YNN	0.0561	0.0000	0.0000	0.0579	–	0.0389	0.0173
IMG	0.0571	0.0059	0.0004	0.0570	0.0023	–	0.0176
HLJ	0.0000	0.1115	0.0866	0.0000	0.1178	0.1181	–

*Values above the diagonal are *G*_*ST*_ and values below the diagonal are *Q*_*ST*_.*

### Isolation-by-Distance in Difenoconazole Tolerance (RGR) and SSR Marker Loci

**FIGURE 4:**
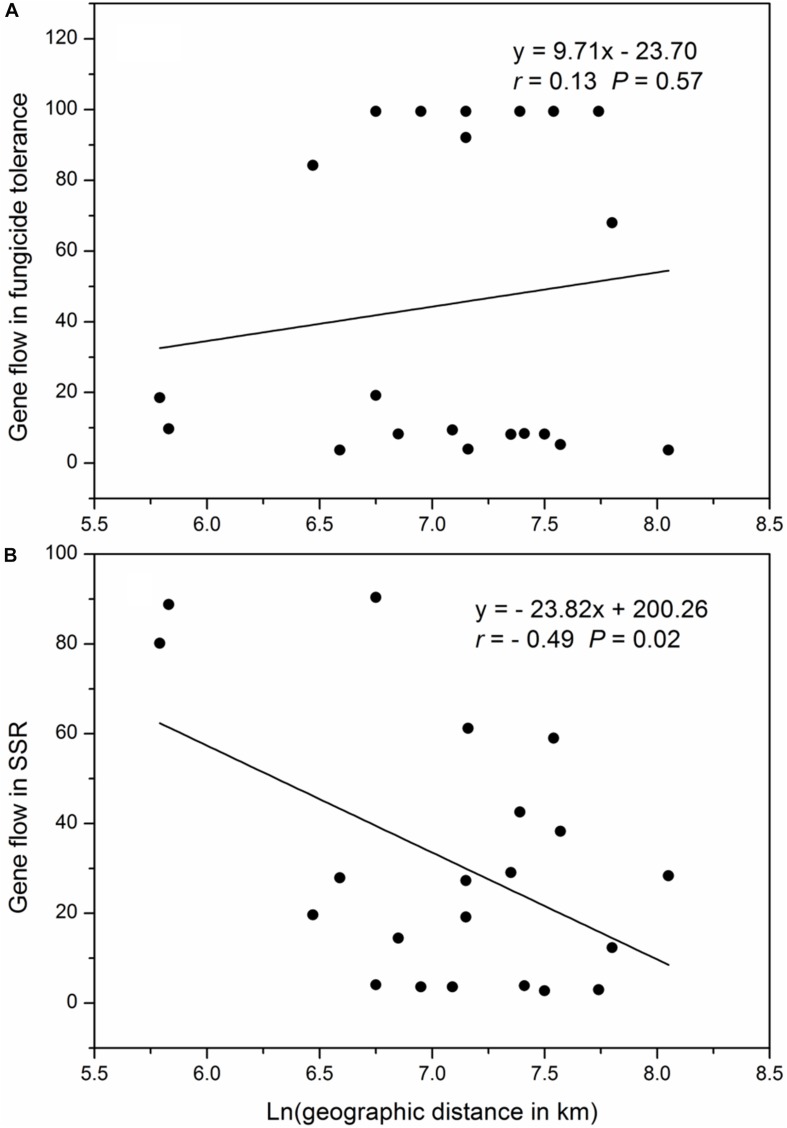
Correlation between Napierian logarithm of the pair-wise geographic distance and pair-wise gene flow among the seven populations of *Alternaria alternata* sampled from potato: **(A)** relative growth rate (difenoconazole tolerance) and **(B)** SSR marker loci.

The geographic distances between pairs of collection sites ranged from 341 to 3146 kilometers, with a mean of 1386 kilometers. The pair-wise gene flow in SSR markers ranged from 3.73 to 99.5 with an average of 45.14 and the pair-wise gene flow in SSR markers ranged from 2.76 to 90.41 with an average of 31.14, There was no correlation between the Napierian logarithm of pair-wise geographic distance and gene flow in difenoconazole tolerance but a significant negative correlation occurred between the Napierian logarithm of pair-wise geographic distances and gene flow in SSR marker loci (Figure 4).

## Discussion

We used a common garden design to investigate the genetics and evolutionary trajectory of fungicide resistance in a large number of pathogen isolates originating from seven distinct regions of China. Our results indicated that the mycelial growth of *A. alternata* isolates was significantly inhibited by small doses of difenoconazole (Figure 2). In addition, no resistant isolates were detected in the widely dispersed pathogen populations sampled despite the continuous and extensive use of the fungicide for several decades in the region (Liang et al., 2009; Chen et al., 2013). Difenoconazole has been used to control many cereal, vegetable and fruit diseases including potato early blight in China since 1998 and ∼10% of its global production is sold in Chinese markets recent years^1^ ^,2^ (Zheng et al., 2013). However, this observation is consistent with previous results showing that difenoconazole is still effective in controlling many plant pathogens such as *A. solani*, *Rhizoctonia cerealis*, *Colletotrichum capsici*, *Didymella applanata*, and *Ceratocystis fimbriata* (Reuveni and Sheglov, 2002; Gopinath et al., 2006; Mirkovic et al., 2015; Wang et al., 2016; Scruggs et al., 2017) and suggests that there is a low risk of developing difenoconazole resistance in many plant pathogens (Hamada et al., 2011; Fonseka and Gudmestad, 2016).

Several factors may contribute to the low risk of developing difenoconazole resistance in plant pathogens. Unlike other site-specific fungicides such as QoI and benzimidazole in which resistance is a qualitative trait and can be quickly developed in pathogen populations (Mcgrath, 2001; Villani et al., 2015; Blake et al., 2018), resistance to DMI fungicides such as difenoconazole is a quantitative trait resulted from the combined effect of: (i) point mutations in the *CYP51* (Cools and Fraaije, 2013; Mair et al., 2016); (ii) overexpression of the *CYP51* enzyme (Ma et al., 2006); (iii) overexpression of genes encoding efflux pump proteins (Price et al., 2015); (iv) alteration in sterol biosynthesis pathways (Karaoglanidis et al., 2003); and (v) changes in pathogen cell wall composition and reduced positive influx (Leroux and Walker, 2011). Evolution of resistance to DMIs, such as difenoconazole, involves sequential accumulation of multiple amino acid substitutions in many independent genes of the pathogen genome. Indeed, using a standard experimental protocol involving a repeated series of infection passaging, mutants that were resistant to some other fungicides but not difenoconazole were detected (Hamada et al., 2011). The finding of a continuous and unimodal distribution in difenoconazole tolerance (Figure 2) accords with the multiple mechanisms involved in the development of difenoconazole resistance.

The low risk of developing difenoconazole resistance in *A. alternata* may also be attributed to the fitness penalty associated with resistance. Previous studies have shown that DMI-resistant mutants exhibited significantly lower fitness compared to their wild-type parents (Karaoglanidis et al., 2001; Reimann and Deising, 2005; Cox et al., 2007; Zhang et al., 2017). In the current study, comparative analysis of spatial distribution in genetic variation indicates the evolution of difenoconazole resistance in *A. alternata* is under constraining selection as demonstrated by a significantly lower population differentiation in difenoconazole tolerance (*Q*_ST_) than in SSR marker loci (*G*_ST_).

In the evolution of fungicide resistance, selection for resistant mutants due to their ability to reduce the efficacy of fungicides or selection against resistant mutants due to severe fitness costs can lead to constraining evolution (Qin et al., 2016; He et al., 2018). We hypothesize that the observed constraining evolution is caused by fitness costs of mutations to difenoconazole resistance because difenoconazole tolerance in *A. alternata* isolates was negatively associated with their aggressiveness though not significantly (data not shown). Constraining selection in the evolution of difenoconazole resistance is also supported by the different patterns of gene flow detected between difenoconazole tolerance measurements and SSR marker loci. Isolation-by-distance was observed in neutral (SSR) loci but not in difenoconazole tolerance (Figure 4), suggesting selection against mutants harmonizes the genetic difference of geographically distant populations accumulated by random drift.

*Alternaria alternata* can infect a wide range of plants including many wild species (Bashan et al., 1991; Thomma, 2003) which are usually not exposed to synthetic fungicides. Previously, we hypothesized that the wild species may serve as a reservoir of pathotypes with high fungicide sensitivity, and that continuous influx of these sensitive pathotypes from wild species prevents or substantially reduces the risk of developing resistance to the non-specific fungicide mancozeb in agriculture (He et al., 2018). This dilution effect through immigrant populations (Andersson and Hughes, 2011) may also explain the low risk of developing difenoconazole resistance in *A. alternata.*

Genetic effects (heritability) account for ∼25% of the phenotypic variation in the tolerance of *A. alternata* to difenoconazole compared to less than 5% attributable to isolate–concentration interactions (plasticity) (Table 3). This result indicates that genetic variation plays a more important role in the evolution of difenoconazole resistance in *A. alternata* than epigenetic variation. This observation is consistent with the positive correlation of difenoconazole tolerance among different concentrations (Figure 3). Heritability leads to lasting adaptation of organisms to environments through changes in gene composition. Plasticity, on the other hand, is a phenomenon whereby a genotype produces different phenotypes through methylation (Angers et al., 2010; Bossdorf et al., 2010), or changes gene expression in response to environmental fluctuations (Winkler and Breaker, 2005; Chen et al., 2015). Unlike other site-specific fungicides that select for strong pathogen–fungicide interactions (i.e., polarized pathogen genotypes that are either highly sensitive or resistant), DMI, as well as non-specific fungicides, selects for pathogen genotypes differing quantitatively in fungicide tolerance. This group of fungicides thus maximizes the accumulation of genetic effect but reduces the effect of particular genotype–environment (concentration) interactions (i.e., plasticity) (He et al., 2018).

Because of their unique mode of action, DMIs are an excellent alternative chemistry and an effective partner to use together with other fungicides to manage plant diseases (Avenot et al., 2016). Although the risk of developing resistance to DMI fungicides is generally low, these fungicides also face resistance problems and cases associated with increased tolerance resulting in reduced or loss of efficacy have been reported for many pathogens (Thomas et al., 2012). For example, it has been reported that *Alternaria* species in California pistachio orchards have become less sensitive to DMIs as a result of regular sprays (Brent and Hollomon, 2007). The finding of significant differences in difenoconazole tolerance among isolates and populations (Table 2) and a skewing of the tolerance distribution toward the right (Figure 2) suggests that a stepwise accumulation of tolerance to difenoconazole might be occurring in the early blight pathogen populations in China. In the study, growth inhibition was only tested in three concentrations due to a large number of isolates. This restriction in dose treatments does not allow us to calculate the half maximum effective concentration robustly (Sebaugh, 2011). A rough estimate found that approximate 1% of *A. alternata* isolates had an EC_50_ value 10-fold greater than the baseline. These isolates have a high potential of developing resistance to difenoconazole in future. Therefore, dynamic management programs formulated by evolutionary principles (Zhan et al., 2014, 2015) such as spatiotemporal rotations of fungicides used alone or in combination with fungicides with different modes of action modes are critical to generate diversifying selection to prevent or mitigate the evolution of resistance to difenoconazole and other DMI fungicides (Valencia-Botín et al., 2013).

## Data Availability

The datasets generated for this study are available on request to the corresponding author.

## Author Contributions

MH-H collected the pathogen isolates, generated and analyzed the data, and wrote the manuscript. Y-PW, E-JW, L-LS, L-NY, TW, L-PS, and WZ generated the data and wrote the manuscript. JZ conceived and designed the experiments, analyzed the data, and wrote the manuscript.

## Conflict of Interest Statement

The authors declare that the research was conducted in the absence of any commercial or financial relationships that could be construed as a potential conflict of interest.

## Footnotes

1 https://www.marketresearchandstatistics.com/ad/difenoconazole-global-product-intelligence/

2 http://www.agrichem.cn/news/2016/3/8/201638135612812.shtml
